# A qualitative study of final-year medical students' perspectives of general practitioners' competencies

**DOI:** 10.5116/ijme.4e79.b49a

**Published:** 2011-09-23

**Authors:** Bjorn Landstrom, Bengt Mattsson, Carl Edvard Rudebeck

**Affiliations:** 1 Department of Public Health and Community Medicine/Primary Health Care Unit, The Sahlgrenska Academy, University of Gothenburg, Sweden; 2 Research Unit, Esplanaden Health Care Centre, Kalmar County Council, Västervik, Sweden and Institute of Community Medicine, Tromsö University, Norway

**Keywords:** General practitioner, medical students, student’s perspective, reflective writing

## Abstract

**Objectives:**

To investigate final-year medical students’ perspectives of general practitioners’ competencies. A further aim of the study was to investigate which type of clinical problems is properly managed by GPs according to students.

**Methods:**

We conducted a qualitative study of 49 final year medical students from two programmes. Reflective writing statements were used to collect data. Qualitative content analysis was employed to analyse data.

**Results:**

Three themes were identified to explain the conditions of a general practitioner (GP). They are: ‘prerequisites’, ‘patients´ problems’ and ‘competence and clinical judgment’ which reflect the specific features of primary care, presentation of symptoms by patient and the way that GPs approach an actual encounter.

**Conclusions:**

The students valued the importance of unselected patient problems, straightforwardness in contact and care as the characteristics of a competent GP. They viewed patients with different approaches and related their observations to problems of fragmentation within this large area of medical care. This is a period in the training of students in which students’ views of general practice are formed.

## Introduction

Over the past ten years, general practice has gained an increasing place in medical training in Europe as early exposure to clinical cases and patient encounter has been emphasised in the undergraduate medical curriculum.^1^^,^^2^ Similar changes in the Swedish medical curriculum have also occurred and students at the six medical schools spend a period of 8-10 weeks in general practice.^3^The World organisation of family doctors (Wonca) has classified core competencies of a GP into six categories. These competencies are: management, patient-centeredness, problem solver, comprehensiveness approach, community-oriented and holistic approach.^4^ These competencies describe a GP’s approach to tasks and patient’s needs. In light of the report by Wonca, the corresponding objectives have been clearly stated in the Swedish Higher Education Ordinance and in medical school curricula.^5^^, ^^6^Medical students’ attitudes towards general practice have been studied in various countries. These showed that students had positive attitudes toward general practice.^7^^,^^8^ One study showed that final year students had more positive attitudes to choosing general practice as a career in the future than first-year students.^9^ Although positive experiences of clinical supervisors in general practice had a clear effect in the short term, negative experiences led to more critical attitudes.^10^ It has also been shown that training courses in general practice has a mostly short-lived effect on students’ behaviour.^11^ The personal enthusiasm of supervisors/GPs can influence medical students towards general practice careers.^12^Despite increased input of general practice into undergraduate medical education, its extent and influence within the whole of the education is still limited.^13^ In addition, hospital attachments dominate and primary care clinicians are rare among the lecturers. Causes, diagnosis and treatment of diseases are mostly taught by preclinical teachers and hospital specialists.^2^ The role of a GP in management and the essential attributes of general practice are seldom addressed in the curriculum.^14^^, ^^15^ There is little information about students’ understanding of general practice at the end of their training. A study reported that students learn primary and community care from different sources, such as witnessing long personal relationships, the effect of social environment on health and dealing with people rather than diseases during their training in health care centres.^16^In Sweden the primary care system is mainly based on publicly-owned health-care centres (HCCs). The residents are in most part of the country registered with a health care centre rather than with a GP. For referrals to other specialists there are regional agreements and the GP does not have a defined gate-keeper role.During the final year of the undergraduate curriculum in Gothenburg the students spend two weeks attached to a GP at a HCC. Students are encouraged to be active in the consultations, visit patients and suggest treatment alternatives even if the GP is formally responsible for the patient’s care. Individual reflections and personal decision-making regarding unselected patient problems are supported. Furthermore a general introduction to primary care is presented. Some students spend their attachment with a GP far away from the university involving daily travelling and overnight stays. GPs voluntarily take responsibility for supervising students. The GPs had attended an introductory course in supervising and they were all interested in teaching and supervising medical students in their consulting rooms.^17^Since there is only scarce research evidence on students’ perspectives of GP’s competencies, we found it important to conduct a qualitative study of this issue at the University of Gothenburg, Sweden. Therefore the purpose of this study was to investigate final year medical students’ perspectives of general practitioners’ competencies. A further aim of the study was to investigate which type of clinical problems students thought were certainly well managed by GPs compared to other specialists.

## Methods

In one introductory day, two weeks before attaching to a HCC, in two successive final-year undergraduate courses in Gothenburg, the students were asked to elaborate on the following task: ‘Describe some situations regarding general problems in general practice where you judge that the GP with his or her competence is specially well equipped to do his or her job compared to other specialists. Explain why you would propose that a GP is suitable to take on the problem. If you find it difficult to call to mind any such situations, describe your thoughts on why this is’. They were asked to make a written commentary to the above statement and return it for follow up two weeks later. The students were free to perform the task and we asked them to express their views by designing 1-2 A4 sheets of papers themselves. Answers were anonymous and the task was not part of the students’ examination. None of the authors were involved in the course management.In the first course the task was voluntary and only 13 out of 36 students submitted their own statements. Therefore in the second course the task was made obligatory and here all students (n=36) submitted their own statements. Consequently 49 students expressed their own views about GPs competencies, on average 1½ A4 pages each. Apart from more or less concrete responses to the task the students commented freely on the issues and clarified their own answers by giving examples.

### Analysis

The scripts of students regarding the task were analysed in several steps using qualitative content analysis.^18^ Initially all authors read the whole material independently to gain a comprehensive picture of scripts. One of the authors (BL) performed the initial coding and later all authors participated for further analysis of data. Preliminary codes and subcategories were identified, compared and discussed. Disagreements were dealt with and the comparisons continued until we agreed on the contents and labels of each subcategory. Finally themes were identified to elucidate the students’ views on the characteristics of general practice.

### Ethical considerations

The task of writing a report was made part of the course. Written and verbal information regarding the study was described and its purpose was explained to the students. Anonymity was warranted and the study was conducted according to general ethical procedure. According to the Regional research ethics committee and Swedish legislation ethical approval is not required for a study of this kind and therefore we did not seek ethical approval.

### Results

Three main themes identified from the students´ scripts were: “Prerequisites”, “Patients´ problems” and “Competence and clinical judgment” (Table 1). These themes reflected and comprised the flavour of GPs’ competence and strengths according to the students. The themes partly reflect a sequence of how the views were presented. To elaborate these themes, first, we describe the specific preconditions of primary care. Second, we explain presentations of symptoms by patients and the way that GPs approach an actual encounter.

**Table 1:** The three themes with categories

Themes	Categories
Prerequisites	- Close to the patient - Co-operation - Close to comfort - Professional lack of inspiration
Patients´ problems	- Unorganized level - Known diseases
Competence and clinical judgment	- The impact of the consultation - Breadth - Whole-person medicine - Continuity

### Prerequisites

#### Close to the patient

From the students’ perspective, GPs examine many patients in order to contribute to the holistic perspective and knowledge of why individuals seek help for their health problems. Knowledge of the local population is an asset in making judgements when changes are taking place. As one of the students reflected:

“...the GP as a warning signal when something is not quite right”.

On the basis of earlier contacts with the patient the physician can also be open to problems of a more private nature hard to approach with less personal knowledge. One student stated:

“…the GP’s forte is to take account of the diversity the patient presents … and to raise rather more personal subjects”.

The GP’s position within health care differs from that of other physicians. He or she is the spider in the net and can help the patient find the right specialist or person. One student reported:

“...help a person find their way within the health services”.

The GP works close to the everyday life of people. This exposes them to occasional and long-term contact, as well as to lesser and greater suffering. Everything is present at the same time, varying throughout the working day. The GP gradually gains enhanced experience of how common complaints appear and learns simple and sometimes standardised modes of action. One of the students stated:

“...violence in the home, graze infections”.

#### Cooperation

According to the students’, the GPs cooperate closely with nurses, physiotherapists and psychologists within the HCC but team-work, accomplished in an organized way, was seldom mentioned. Collaboration with representatives of the community and contact with municipal employees and social workers was also mentioned as important. The GPs have favourable prerequisites for these connections compared to hospital physicians. In palliative care home visits are common and their implementation requires the GP’s ability to cooperate. One student commented:

“…the GP often has deeper knowledge of how cooperation with the municipality, social services and so on functions”.

#### Close to comfort

Good accessibility for patients was stressed in the students’ reports. Particularly uneasy patients can get an appointment promptly. Even if they do not always visit, the possibility is there. The patient knows that comfort is close. As one student pointed out:

“...the patient knows where to turn”; "somewhere you can make an appointment”.

Unclear symptoms can also be investigated without too much hurry unless medical reasons dictate haste. Another student suggested:

“...the investigation can be more gradual, the patient has time to think the situation over”.

The students’ had learnt that good accessibility also involves simple, uncomplicated routines at the HCC without any certain requirements for acceptance. A contact with the patient can be maintained in a natural way without special reasons, and a follow-up is easily achieved. A satisfactory accessibility at the HCC was also pointed out, a striking comparison with telephone accessibility at hospital was mentioned. One student put it:

“...re-visits easier, possibilities for good honest meetings”.

Uncomplicated work routines also make the HCCs more homelike than hospitals. The atmosphere is reasonably informal, especially favouring patients with experiences of psychiatric settings. For instance one student stated that:

“…the surroundings are less trying for psychiatric patients”.

#### Professional lack of inspiration

The students’ reports contain minor comments about limitations and shortcomings in working conditions. However, the GP’s limited opportunities for further training and professional exchange of ideas and inspiration were pointed out. Working on one’s own gives less opportunity for exchange of experiences and further training compared with what the hospital can offer as reflected by one student:

“...hospital staffs have the advantage that they can meet and train each other”.

### Patients’ problems

#### Uncertainty level

Students stated that the GP is the first physician to meet the patient’s symptom presentation. Yet a nurse might have made an earlier assessment and a certain categorisation may already be at hand. Separating the trivial from the serious was described as a characteristic task in general practice. Many patients worry that their symptoms are a sign of a serious illness and want a doctor’s judgement. A more general diagnosis is made at the HCC and often there is no reason to obtain a more specific diagnosis. One student commented:

“...patients may say ‘I don’t think there’s anything seriously wrong with my knee, I’d just like you to check it’”.

The students had observed that to grasp the patient’s symptoms the GP often uses several stages of approaching the cause and examinations are done more gradually. No complete examination or numerous laboratory tests are carried out from the start and a variety of organ systems are considered. The focus of the examination is more directed at consequences than at organs and diagnosis. One student reflected*:*

“… the GP may say, ‘now we’ll screen for what can be dangerous’”.

The GP is early confronted with patients’ fear and anxiety in relation to new symptoms. Together with the patient the GP could find out underlying causes of stressors and give it a proper meaning. In this regard one student asserted:

“See whether there is anything else that the patient is worried about”.

Some students, however, also stated that the GP’s contribution to health care is limited. The GP’s main task was presented as sorting, giving health advice, tidying up among drugs and writing referrals. For example one student commented:

“…the doctor as a sorting machine”.

#### Known diseases

GPs can thoroughly manage patients with chronic diseases. They also managed patients with specific diseases in different organ systems that needed regular visits. Focusing on patient care was also stressed. For instance one student stated:

“…while not so serious individually the diseases together become a problem that limits the patient’s daily life”.

In this context students also reported that GPs economically contribute to the medical care system in the way that one student stated:

“…GPs greatly relieve the pressure on hospitals”.

### Competence and clinical judgement

#### The impact of the consultation

The students described the consultation in concrete terms; the meeting between the patient and the physician. Often the encounter was characterized as the GP having a firm and concrete grip on things. Experiences of active and work-intensive hospital consultations where a multitude of facts are gathered and organised was contrasted with the physician’s work at HCC. One student stated:

“…even in short consultations problems can be identified thanks to knowledge of the patient”, “…interesting to see that the doctor did not need to do so much: listening was enough”.

The students described ability at consultations as a kind of open mindedness with regard to surprises and the ability to achieve spontaneity. For instance one student reflected:

“…the ability to be receptive and sensitive to confront fear in the patient”.

Additionally, in terms of GP-patient relationship students seldom stated that time constraints were an issue.

In many students’ eyes the GP possessed a special talent for talking and making contact with the patient in a relaxed manner trained or achieved in some other way. As a consequence the patient can be in a more equal position. One student commented:

“...the individual was able to speak”; “…the GP is perhaps more than anyone else a doctor for the soul”.

#### Breadth

In the students’ opinions the GP has special scope for lateral thinking and can make choices that the hospital physician might find difficult as being incompatible with his/her position. The students noted that the GP has a generalist competence, an ability that embraces what is in common beyond the particular ways individuals and diseases express themselves.

“...generalism as the ability to recognise complex patterns within health”; “...gain enormous experience of how different disorders normally appear”.

Having knowledge available within different disciplines enables GPs to alter their own focus on patients and sometimes helps GPs to discover or identify the exact cause of an illness or a problem. One student commented:

“...an orthopaedic problem can be a mental problem”; “...the patient does not need referring to different specialists”.

Student reported that the GP has some degrees of freedom for action and a greater chance for assessing patients' needs on an individual basis. One student put it:

“...combining tested experience with one’s own experience in any given case”.

One student clearly linked competence with experience within a broad field:

“...the ability to sift out what is less important from what is important, presumably because they see such a large number of patients with different complaints and symptoms”, “….gaining in the course of time a very broad knowledge base which I think many specialists lose after a time.”

Patient groups with no obvious place in specialised care, often with appreciable and protracted suffering, were found by the students to be well suited to the GP, for reasons of competence and organisation alike. Some of the students coined the term “mildly-psychiatric” patients, meaning patients with anxiety and depression, not qualified for specialist psychiatric care. The patients themselves do not describe their disorders as mild. One student commented:

“...easier to handle psychiatric problems …have time to build a good relationship with the patient.”

GPs are also more ‘harmless’ than psychiatrists:

“...the stigma attached to an actual psychiatrist.”

Patients with problems of abuse also constituted a group for which the students stressed the importance of patient knowledge and opportunities for follow-up. For example one student reported:

“...creating good relationships and learning more of the patient’s life situation”.

The students also noticed the possibility for prevention of disease at an early stage. When continuity was at hand the patient often gained a readiness to accept opinions on his or her way of living. One student reflected:

“...can start by talking about lifestyles”

Many investigations could end at an early stage. Quite early on in a consultation the GP’s clinical experience, or a basic examination, could support the opinion that most likely a serious disease was less probable. One student stated:

“...there is half-screening”.

#### Whole-person medicine

The term “holistic view” was used by several students without a clear explanation. The holistic approach is partly linked to the notion of breadth, yet in the reports the term can be distinguished as a special ability to perceive the whole picture rather than to possess a great knowledge. A general practice attitude stresses more the whole than a separate medical finding. The GP is sensitive to the patient’s needs more clearly than the specialist and gives support differently. For instance one student reported:

“...the GP who with his knowledge of the patient and his overall view can better advise the patient as to whether he should have a prostate operation than the urologist can”.

The students described this holistic approach as a humanistic attitude, an ability to “see the person”, to see the individual, whether he or she is ill or only thinks so. One student put it:

“...treating the person rather than the illness”.

The GP also has an ability to think comprehensively about health problems. This is sometimes related to the drawbacks of specialisation. One student reflected:

“...I feel instinctively that a patient has a complexity of health problems that is not limited to one problem”. “...the GP meets the patient without the tunnel vision one tends to develop as a specialist”.

Possessing an overall view was also described as a necessary preparation for being able to give support when the patient’s existence is being seriously threatened by a severe disease. One student stated:

“...the need of developed trust to be able to handle a new and more serious disease”.

#### Continuity

In the students’ view there is a context in the surroundings, in the broad sense, in which the patient lives. One student reported:

"...the patient one day, her husband the next, and then we understood more”.

The students also stressed the importance of continuity, and continuity had many aspects. Knowledge of the patient’s background makes it easier to make an initial assessment. For example one student put it:

“...evaluating new symptoms starting from a baseline”.

By regularly seeing patients it is also easier to obtain an understanding of the symptoms presented. Continuity created connections as one student stated that:

“...you get an explanation of and understanding of the cause underlying the symptoms”.

Considering the GP work over a longer time period makes it possible to gain feedback on health-preventive inputs and the GP has the potential for health-oriented work. One student reflected:

“...has different time parameters for tackling those worrying problems and can thus contribute greatly to popular wellbeing”.

In having frequently recurring visits by patients or their relatives the GP also gains an impression of the context of a patient in a broader sense. Considering the surroundings may enhance the clinical view.With temporary locums, present in some HCCs, the students were aware of the lack of continuity in the system. It seems that the benefits of primary care are not fully exploited by locums as one student stated:

“...thought there would be many locums who did not care about the patients”.

## Discussion

We asked final year medical students in two courses to write about situations they had experienced concerning the competencies of a GP. The students’ reports were rich in content and the impression they expressed the students’ own views, and not just those of their teachers, was strengthened by the fact that many of the reports opened with authentic scenes from practice. In most respects the reports were consistent with the declaration of the established organisations (Wonca, EURACT) issue on GP competences. Unselected patient symptomatology, “medical breadth” and a holistic approach were often described as characteristics of this mode of work. Access to a GP is based on simplicity and the GP is extremely well placed to manage “everyday suffering”. The terms “whole person medicine” and “continuity” recurred frequently.Students reported that ‘medical breadth’ has several dimensions; it covers the management of several different diseases and possession of experience of how different symptoms are manifested. Breadth also comprises the ability to think broadly in the process of diagnosis. The students described the GP as firm and down-to-earth *“...the GP fixes most things”*. Breadth affords a sort of accuracy. It seems that the students viewed the GP’s broad practical experience as an important component of their competence. The same conclusion emerged in our earlier study when the GPs themselves described their competence.^19^One student commented a striking description of the usefulness of on-going unselected patient contact*:*

“The ability to sift out the less important from the important, presumably because they see such a large number of patients with different complaints and symptoms.” “…They gain over time a very broad base of knowledge which I think many specialists lose in time”.

The students appeared to have a self-evident perception of the holistic approach, expressed as *“treating people rather than symptoms”*, which is in accordance with the definition of the Wonca.^4^ But despite the holistic approach the students wrote little about patient-centred care and what the patients want. The patient’s medical problems were described by the students comprehensively but most often with the patient as someone who needed taking care of and very seldom as an acting subject. A familiar paternalistic perspective still appears to hold sway.^20^Conspicuously common were comments on the patient’s ease of access to care. The students stressed the simple way general practice is managed, based on knowledge and geographical proximity. This indicates that the students had a traditional view of the GP’s mode of work. Olesen and colleagues asserted that GPs ways of working would change. They would lose the total responsibility and instead take a role of consultant in the different fields of health care.^21^GPs’ prerequisites also include being able to manage everyday medical care, and here particularly the students focused on “everyday suffering”. The suffering occasioned by chronic pain, chronic diseases and everyday anxiety, in which other specialists appear not to be particularly interested, or even equipped to bother about. The students used expressions such as; “time to explain”, “several diseases together a problem” and “find out more about the life situation”. Again the students granted general practitioners time and the opportunity to tackle this everyday suffering with a special competence.Some of the student formulations, about sorting and health care, gave the impression that they did not allow GPs any distinctive importance. One student stressed that hospital physicians have better opportunities to talk to one another and that the GP finds it difficult to maintain his/her competence. Embedded in that assertion is the notion that general practice is an activity in which development stagnates because one works too much on one’s own.Remarkably little was written about the GP’s limitations. This may partly be ascribed to a wish not to expose a critical attitude. But it is more likely that this meagreness had to do with the orientation of the question towards the GP’s special competence. The scarcity of descriptions of time-constraint might be explained by that time per consultation tends to more generous when students visit the HCCs, and it may also be that the GPs manage to hide their feelings of stress to the students.There were few descriptions of co-operation. A possible explanation is that the students have not observed all that much co-operation, which could suggest that co-operation in the direct sense, does not represent a very large part of the GP’s time.^22^ On the contrary an English study emphasized the use of the whole team for care.^16^Despite an increasing proportion of general practice input, medical students are for the most part trained in hospitals by various sub-specialists. For this reason it was encouraging to find how markedly positive, enthusiastic and nuanced the students’ attitudes were. The students identified general practice as a speciality clearly and positively which is in line with the few other studies in the field that we have found.^16^^, ^^23^ It may be that the enthusiasm of some curriculum planners for an expanding general practice component in their training, and particularly the interested and experienced GP supervisors, affected the students’ attitudes positively? ^12^^, ^^24^

### 

#### The limitations of the study

The study was performed in Gothenburg, Sweden, and it is not possible to judge the transferability of the results. However, the fact that patient encounter is put in focus in medical education internationally suggests that our encouraging findings may also apply in other settings where GPs are involved on a broader scale of training. Factors that limit transferability are the differences among the systems of primary care, and of their role in the whole of health care, as well as the differences in medical education among universities and countries. Since the reports were anonymous, it was not possible to take gender into account. In a German study^25^ women appreciated general practice in certain aspects more than men. Further studies about the student perspective on GP’s competence might challenge our results in interesting ways.

## Conclusions

The encouragement of active, reflecting and decision-making students in the final year of the undergraduate curriculum in Gothenburg is made explicit in a many-facetted apprehension of GP competence as perceived by the students. Reflective writing statements indicate that, albeit brief, students’ experience was of considerable importance. From their striking, personal and markedly positive descriptions there emerges a profession that has much in common with the Wonca statement published in 2005. The students reported as typical for general practice: unselected patient problems and simplicity in contact/care with the patients.The medical students view people from an existential, bio-psychosocial perspective. They view problems in the disintegration in large sections of medical care and stress a holistic perspective on general practice. Thus it appears that during the final year of the programme, the understanding of, and confidence in general practice is considerable. It is important to conduct further studies on how to build this confidence in order to increase the number of doctors choosing general practice as a career.

## 

### Conflict of Interest

The authors declare that they have no conflict of interest.
